# E-Liquid Containing a Mixture of Coconut, Vanilla, and Cookie Flavors Causes Cellular Senescence and Dysregulated Repair in Pulmonary Fibroblasts: Implications on Premature Aging

**DOI:** 10.3389/fphys.2020.00924

**Published:** 2020-09-04

**Authors:** Joseph H. Lucas, Thivanka Muthumalage, Qixin Wang, Michelle R. Friedman, Alan E. Friedman, Irfan Rahman

**Affiliations:** ^1^Department of Environmental Medicine, University of Rochester Medical Center, Rochester, NY, United States; ^2^Department of Chemistry & Biochemistry, The College of Brockport, The State University of New York, New York, NY, United States; ^3^Department of Materials Design and Innovation, School of Engineering and Applied Sciences, University at Buffalo, Buffalo, NY, United States

**Keywords:** e-cigarette, e-liquid, senescence, wound healing, extracellular matrix

## Abstract

Electronic cigarette (e-cig) usage has risen dramatically worldwide over the past decade. While they are touted as a safe alternative to cigarettes, recent studies indicate that high levels of nicotine and flavoring chemicals present in e-cigs may still cause adverse health effects. We hypothesized that an e-liquid containing a mixture of tobacco, coconut, vanilla, and cookie flavors would induce senescence and disrupt wound healing processes in pulmonary fibroblasts. To test this hypothesis, we exposed pulmonary fibroblasts (HFL-1) to e-liquid at varying doses and assessed cytotoxicity, inflammation, senescence, and myofibroblast differentiation. We found that e-liquid exposure caused cytotoxicity, which was accompanied by an increase in IL-8 release in the conditioned media. E-liquid exposure resulted in elevated senescence-associated beta-galactosidase (SA-β-gal) activity. Transforming growth factor-β1 (TGF-β1) induced myofibroblast differentiation was inhibited by e-liquid exposure, resulting in decreased α-smooth muscle actin and fibronectin protein levels. Together, our data suggest that an e-liquid containing a mixture of flavors induces inflammation, senescence and dysregulated wound healing responses.

## Introduction

Electronic cigarettes (e-cigs) have become increasingly popular in western countries, particularly among adolescents. These devices generate aerosols from refill liquids (e-liquids) containing nicotine and flavoring compounds solubilized in a humectant such as propylene glycol (PG) or vegetable glycerin (VG) (Barrington-Trimis et al., 2014; Goldenson et al., 2017). E-cigs have been marketed as a safer alternative to conventional cigarette smoking, but the availability of flavored e-cigs has led to an epidemic of nicotine addiction among teenagers. E-cig use among high school students nearly doubled from 11.7% to 20.8% during the 2017–2018 period (Gentzke et al., 2019), and recent efforts to limit use among younger users have led to a federal ban on the sale of prefilled cartridges with flavors except for menthol and tobacco in the United States (FDA, 2020). However, consumers may still fill their own cartridges or transition to other flavored tobacco products (Yang et al., 2020). Flavoring chemicals such as vanillin often contain aldehydes, which are known to cause DNA damage and senescence, markers of aging (Sundar et al., 2016). While cigarette smoke is an established driver of premature aging (Koh et al., 2002; Garcia-Arcos et al., 2016; Vij et al., 2018), there is little information on the effects of e-cigarettes on aging.

Aging is defined as the progressive deterioration of physiological functions over time (Meiners et al., 2015). These changes are accompanied by increased inflammation, dysregulated repair processes, and senescence, a state of irreversible growth arrest. The lung is constantly exposed to environmental challenges such as cigarette smoke, fumes, pollen, and viral and bacterial pathogens, which are normally cleared by specialized immune cells (Meiners et al., 2015). When there is sustained long term exposure to contaminants such as cigarette smoke, the defense systems in the lung can become overwhelmed leading to deleterious structural alterations. Cigarette smoke is thought to accelerate these changes by inducing oxidative and DNA damage responses in pulmonary fibroblasts resulting in stress-induced senescence (Nyunoya et al., 2006; Miglino et al., 2012). Fibroblasts are mesenchymal cells that help maintain the extracellular matrix (ECM), a complex meshwork of fibrous proteins, glycoproteins, and proteoglycans that provide scaffolding and structural stability in the lung (Chilosi et al., 2012). Senescent fibroblasts accumulate in older individuals and are thought of as a defense mechanism to prevent dysfunctional or potentially tumorigenic cells from continuing to proliferate (Baraibar et al., 2012; Rashid et al., 2018). However, senescence may prevent fibroblast proliferation during wound healing and these cells also adopt a senescence-associated secretory phenotype (SASP), releasing proteases, growth factors, and proinflammatory mediators/cytokines (Lerner et al., 2015b; Sundar et al., 2016) that maintain a proinflammatory phenotype which may further predispose the lung to age associated pulmonary exacerbations.

Another consequence of aging is the inability to maintain proper repair processes in the lung. These changes can lead to a further decline in pulmonary function (Meiners et al., 2015). During wound healing, pulmonary fibroblasts migrate and differentiate into myofibroblasts, the main effector cells that regulate the production and organization of the ECM. These effector cells secrete ECM proteins that serve as scaffolding for epithelial cells migrating into the wound (Ko et al., 2019). Once wound resolution initiates, myofibroblast undergo apoptosis. However, in interstitial lung disease (ILD) and aged lungs, these myofibroblasts are apoptosis-resistant (Huang et al., 2015; Hanson et al., 2019), leading to abnormal ECM accumulation. Myofibroblast differentiation is primarily controlled by the cytokine, transforming growth factor-β1 (TGF-β1) (Sandbo et al., 2009). Studies show that nicotine and potentially other e-cig constituents can inhibit TGF-β1 signaling, perturbing wound healing processes in the lung (Silva et al., 2012; Lei et al., 2017).

In this study, we assessed the effects of a commercially available e-liquid, a mixture of coconut, cookie, and vanilla flavors containing nicotine (3 mg/mL) on inflammation and senescence in pulmonary fibroblasts. Furthermore, we hypothesized that e-liquid exposure would disrupt myofibroblast differentiation, revealing undesired alterations to cell physiology that would be consistent with accelerated aging. Our previous work shows that chronic e-cig users have increased inflammatory and oxidative stress biomarkers. E-cig generated aerosols were also shown to contain comparable levels of reactive oxygen species (ROS) to cigarettes (Lerner et al., 2015a,b). E-cig exposure can also induce DNA fragmentation which can lead to senescence in human lung cells (Lerner et al., 2015b). However, there is little information available on the effects of e-cig flavorings on lung cellular senescence. E-cigs contain flavoring chemicals, humectants, and often nicotine. Since humectants and flavoring additives are generally recognized as safe (GRAS) in foods, it has been wrongly assumed that these compounds would be innocuous when inhaled (Sears et al., 2017). However, our recent work, as well as others, show that flavoring compounds, such as vanillin and cinnamaldehyde, induce oxidative stress and inflammatory responses in human lung cells (Hua et al., 2019; Muthumalage et al., 2019). Furthermore, PG and VG, two common e-cig vehicles, may alter extracellular matrix remodeling and inflammatory-immune responses in the lung (Madison et al., 2019; Wang et al., 2019) consistent with the promotion of aging. It is possible that the e-liquid containing flavoring chemicals may induce pro-senescence and dysregulated repair responses.

## Materials and Methods

### Scientific Rigor

We used a rigorous and unbiased approach during experiments and data analysis.

### E-Liquid Mixture of Flavors/Flavoring Chemicals

The e-liquid, a mixture of tobacco, coconut, vanilla, and cookie flavors, was kindly provided by the Belgium Ministry of Public Health. It consists of 50/50 PG/VG, nicotine (3 mg/ml), and tobacco, coconut, cookies, and vanilla flavors.

### Gas Chromatography and Mass Spectrometry

Gas chromatography and mass spectrometry (GC-MS) was carried out as previously described (Muthumalage et al., 2019).

### Cell Culture and Treatment

Human lung fibroblasts cells (HFL-1) were purchased from ATCC (Manassas, VA, United States) and cultured in Dulbecco’s Modified Eagle’s medium (Gibco; #10569-010, Carlsbad, CA, United States) and supplemented with 1% of penicillin/streptomycin (Gibco; #15140-122), 1% non-essential amino acids (Gibco; #11140-050), and 10% fetal bovine serum (FBS). Cells were treated with various doses of flavored e-liquid (0.1–1% v/v), nicotine (Sigma Aldrich; #200-607-2), 50/50 PG/VG^1^, and/or 5 ng/mL TGF-β1 (ab50036) for 24 or 72 h. HFL-1 were between passages 6–10 and cultured at 5% CO_2_ at 37°C in T75 flasks.

### Cell Viability and ELISA

HFL-1 (5 × 10^4^ cells/well) were cultured in 24 well plates to 80% confluency and serum-deprived overnight in 1% FBS. Cells were lifted with 0.25% trypsin with EDTA following treatment and neutralized with complete medium. Cells were stained with Viastain^TM^ AO/PI (Nexelcom Biosciences; #CS2-0106, Lawrence, MA, United States) and counted on a Cellometer Auto 2000. IL-8 release was measured in cell supernatants using an IL-8 Human Matched Antibody Pair Kit (Invitrogen; #CHC1303, Carlsbad, CA, United States) according to the manufacturer’s instructions.

### Western Blotting

Protein concentrations were measured in whole-cell lysates by Pierce BCA Protein Assay Kit Thermo Scientific; #23225, Waltham, MA, United States). 10 μg protein was separated in 10% SDS-PAGE gel and transferred on to a nitrocellulose membrane. The membrane was incubated with anti-α-SMA antibody (ab124964, 1:1000), anti-Fn antibody (ab2413, 1:1000), and anti-Col1A1 antibody (ab21286, 1:1000) from abcam (Cambridge, MA, United States) overnight at 4°C. The following day, the membrane was incubated with HRP-conjugated secondary anti-rabbit antibody (BioRad; #170-6515, 1:5000) for 1 h at room temperature. The chemiluminescence was detected using the Bio-Rad ChemiDoc MP imaging system. Densitometric analyses of the band intensities were performed using Image Lab software (v4.1, BioRad, Hercules, CA, United States). GAPDH (ab9484, 1:2000) was used as the endogenous control for normalization.

### Cellular Senescence Activity Assay

Detection of SA-β-gal activity was determined by the conversion rate of 4-methylumbelliferyl-β-D-galactopyranoside (MUG) to the 4-methylumbelliferone (4-MU) using a kit (ENZO; #130-0010, Farmingdale, NY, United States). The assay protocol was adapted from the manufacturer’s instructions. Briefly, 50 μL of protein (20× dilution) was added to 50 μL of 2× assay buffer (40 mM citric acid, Na_3_PO_4_, 300 mM NaCl, 10 mM β-mercaptoethanol, 4 mM MgCl, and 1.7 mM MUG at pH 6.0) and incubated for 3 h. 50 μL of the solution was transferred to another plate and 200 μL of stop solution was added. Senescence activity was defined as the fluorescence intensity at 360 nm excitation and 465 nm emission. Data were normalized to protein concentration.

### Statistical Analysis

Statistical analyses of significance were performed by one-way ANOVA followed by Tukey’s multiple comparison test when comparing multiple groups using GraphPad Prism 7 (La Jolla, CA, United States). Data are presented as means ± SEM. *p* < 0.05 is considered as statistically significant.

## Results

### Mixed Flavored E-Liquid Induces Cytotoxicity in HFL-1 Fibroblasts

**FIGURE 1:**
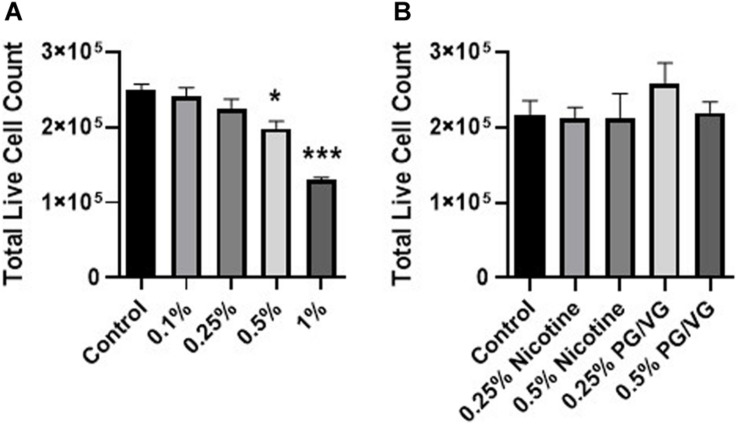
Mixed flavored e-liquid induces cytotoxicity in HFL-1. Total live cell counts of a **(A)** mixed flavored e-liquid, **(B)** nicotine or PG/VG controls in HFL-1 after 24 h exposure. Cells were stained with AO/PI dye and counted on a Cellometer Auto 2000. Data represented as mean ± SEM (*n* = 4 per group). *p ≤ 0.05, ***p ≤ 0.001 vs untreated control group. Only significant differences vs control were labeled with asterisks.

To investigate the cytotoxicity of the e-liquid flavors, HFL-1 cells were exposed to concentrations between 0.1 to 1% v/v for 24 h. A 50/50 mixture of PG/VG and nicotine controls were included in the study. E-liquid exposure demonstrated significant cytotoxicity at 0.5 and 1.0% concentrations. Total live cell counts were 79.6 and 52.0% relative to control for 0.5 and 1.0% concentrations, respectively. There was no associated toxicity with equivalent PG/VG or nicotine controls for 0.25 and 0.5% dose (Figures 1A,B).

### Inflammation and Cellular Senescence in HFL-1 by Mixed Flavored E-Liquid

**FIGURE 2:**
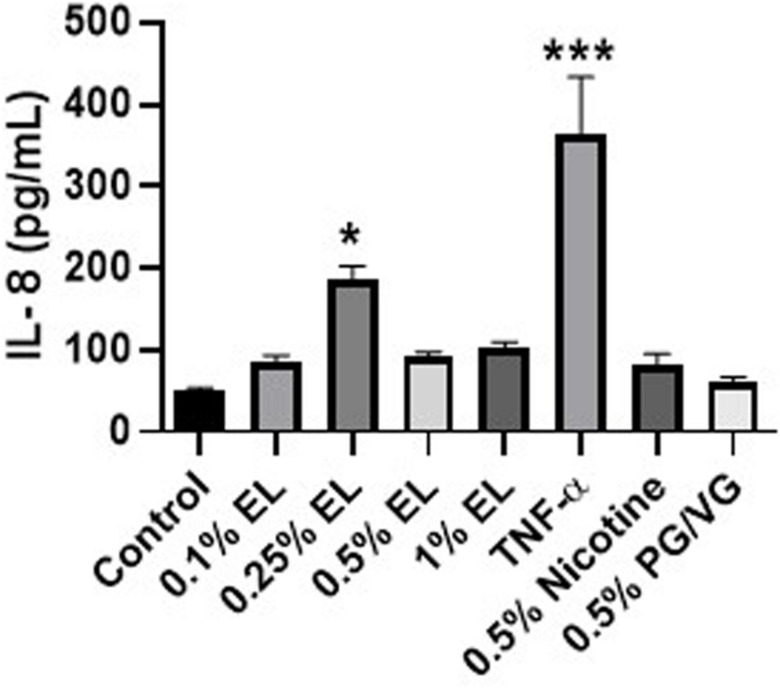
Mixed flavored e-liquid induces an inflammatory response in HFL-1. Cells were exposed to e-liquid (EL), nicotine, PG/VG, or 10 ng/mL TNF-α for 24 h. IL-8 release was measured in the conditioned media following exposure by ELISA. Data represented as mean ± SEM (*n* = 4 per group). *p ≤ 0.05, ***p ≤ 0.001 vs untreated control group. Only significant differences vs control were labeled with asterisks.

To determine if a mixture of flavors elicited an inflammatory response, we exposed HFL-1 to various doses of e-liquid with the appropriate PG/VG and nicotine controls. TNF-α was used as a positive control and indicates that the cells were responsive to proinflammatory stimuli. IL-8 was measured in the conditioned media 24 h post-treatment. IL-8 release was significantly elevated at 0.25% e-liquid. However, at higher concentrations, IL-8 release was unchanged compared to control. There was no change in IL-8 release in nicotine or PG/VG treated cells compared to control (Figure 2).

**FIGURE 3:**
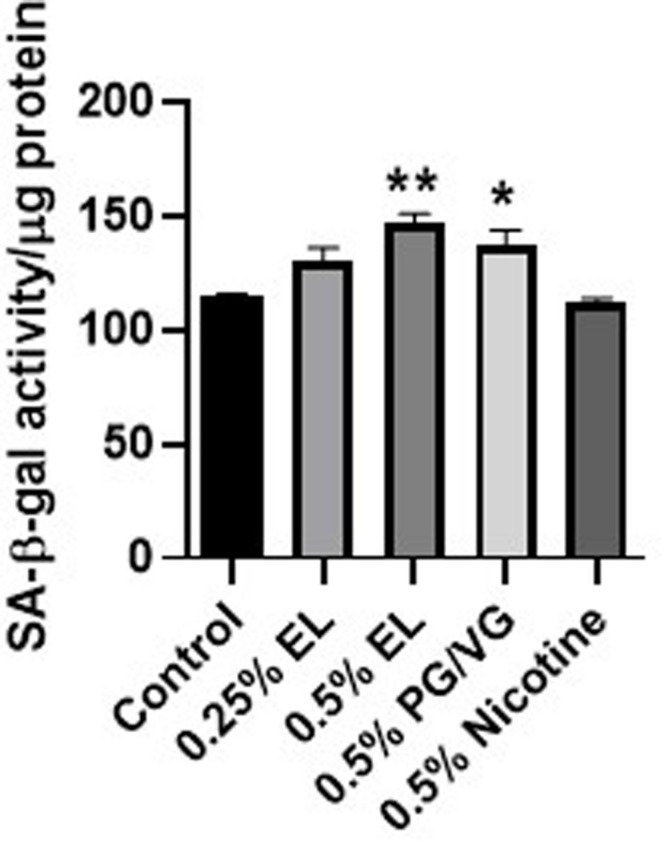
Cellular senescence was caused by a mixed flavored e-liquid in HFL-1. Cells were treated with e-liquid (EL), nicotine, or PG/VG for 72 h. Cells were lysed and SA-β-gal activity was assessed by measuring the conversion rate of 4-MUG to 4-MU. Data represented as mean fluorescence intensity normalized to protein concentration ± SEM (*n* = 3 per group). *p ≤ 0.05, **p ≤ 0.01, vs untreated control group. Only significant differences vs control were labeled with asterisks.

Senescence was assessed in HFL-1 exposed to varying concentrations of e-liquid for 72 h. SA-β-gal activity was measured as a marker of cellular senescence in these cells. Exposure to 0.5% e-liquid and PG/VG showed a significant increase in SA-β-gal activity compared to controls. There was no effect with nicotine treatment (Figure 3).

### E-Liquid Inhibited TGF-β1 Induced Myofibroblast Differentiation

**FIGURE 4:**
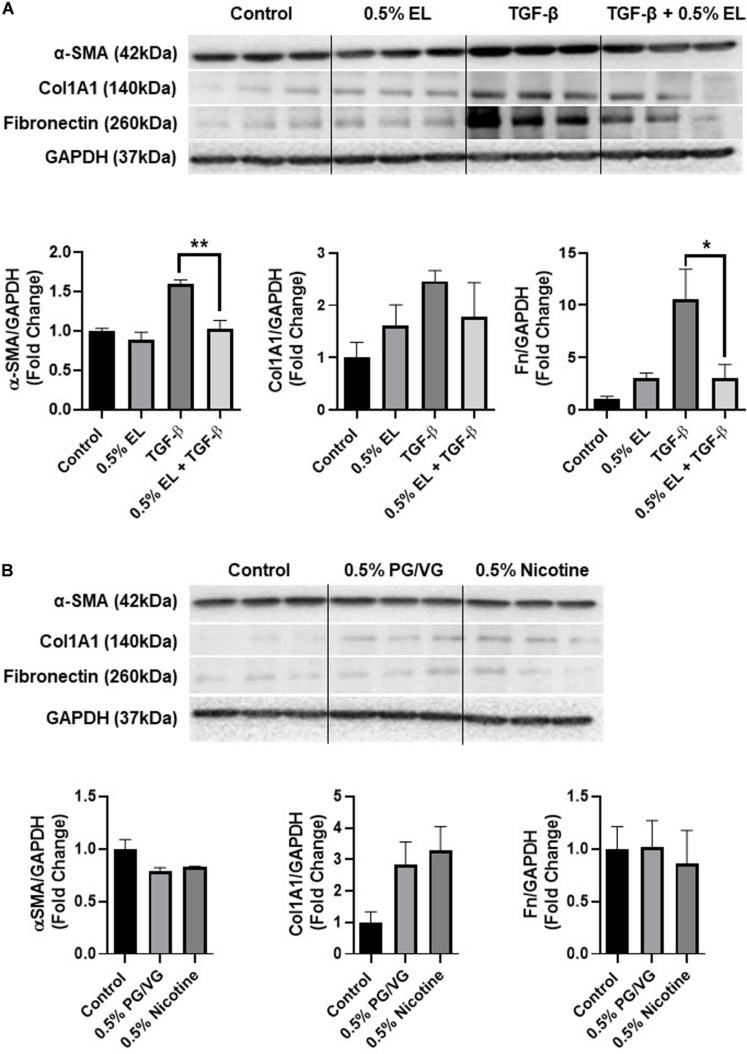
E-liquid inhibited TGF-β1 induced myofibroblast differentiation. Immunoblots following exposure to a **(A)** mixed flavored e-liquid (EL) and/or 5 ng/mL TGF-β1 after 72 h or **(B)** nicotine and PG/VG controls are shown. The protein abundance of extracellular matrix related markers was measured in whole-cell lysate using western blotting. GAPDH was used as an endogenous control. Representative blots for α-smooth muscle actin (α-SMA), Fibronectin (Fn), and type I collagen (COL1A1) in HFL-1 are shown. The band intensity was measured by densitometry and data are shown as fold change relative to control. Data are shown as mean ± SEM (*n* = 3/group) *p < 0.05, **p < 0.01, indicates significance. Only significant differences vs control were labeled with asterisks.

Inhalation of toxic substances can cause damage to the lung and initiate wound healing responses. The production of ECM proteins was assessed by immunoblot analysis in response to e-liquid exposure alone and in combination with 5 ng/mL TGF-β1. Protein levels of α-SMA, a marker of myofibroblast differentiation was assessed. E-liquid exposure did not alter levels of α-SMA after 72 h compared to control. However, e-liquid exposure did significantly prevent TGF-β1 induced myofibroblast differentiation, measured by α-SMA levels. When we analyzed the production of ECM proteins, e-liquid treatment did not significantly alter levels of fibronectin or type I collagen. However, inhibition of TGF-β1 induced fibronectin was observed (Figure 4A). There were no significant changes in PG/VG or nicotine exposed groups (Figure 4B).

### Characterization of Chemical Constituents Contained in Mixed Flavored E-Liquid

The constituents of the mixed flavored e-liquid were categorized broadly into known flavoring additives, silicon-containing compounds, humectants and oils, terpenes, alkanes, and miscellaneous in Table 1. The predominant flavoring constituents were pyrazines, vanillin, and furonones.

**TABLE 1:** Constituents detected in e-liquid cartridge by GC-MS.

Flavoring chemicals	Humectants/solvents	Silicon compounds	Terpenes	Alkanes	Miscellaneous
benzaldehyde, 3,4- dimethoxy-, methylmonoacetal-	heptaethylene glycol	1-methoxy-5-dimethyl(ethyl)silyloxy-3-phenylpentane	cis-beta-terpineol	tetradecane, 2,6,10-trimethyl	4,5-dihydro-4,4-undecamethylene-2-phenyl-1,3-oxazin-6-one
pyrazine,2,3-dimethyl	glycerin	1-butyl(dimethyl)silyloxypropane	cyclohexanol, 1-methyl-4-(1-methylethyl)-	octadecane, 3-ethyl-5-(2-ethylbutyl)-	6,7-epoxypregn-4-ene-9,11,18-triol-3,20-dione, 11,18-diacetate
pyrazine, trimethyl	methoxyacetic acid, 2-tetradecyl ester	silane, diethoxydimethyl-	squalene	tetradecane, 2,6,10-trimethyl-	butanedioic acid, 2,3- dimethoxy-, diethyl ester
2(3*H*)-furanone, 5-heptyldihydro-	9-octadecenoic acid (Z)-, methyl ester	diisopropyl(ethoxy)silane	–	–	butanoic acid, 4-(1,1-dimethylethoxy)-3- hydroxy-, methyl ester, (R)
2-cyclopenten-1-one,2-hydroxy-3-methyl	10-octadecenoic acid, methyl ester	cyclohexasiloxane, dodecamethyl-	–	–	3-ethoxy-1,2-propanediol
1,2-cyclopentanedione,3-methyl	octadecanoic acid, methyl ester	4-methyl(trimethylene)silyloxyoctane	–	–	urea
menthol	10-octadecenoic acid, methyl ester	cycloheptasiloxane, tetradecamethyl-	–	–	teredphthalic acid, 2-nitro-5-sulfanyl-
2(3*H*)-furanone,5-butylhydro-	octadecenoic acid, methyl ester	cyclononasiloxane, octadecamethyl-	–	–	dithiocarbamate,5- methyl-,*N*-(2-methyl-3-oxobutyl)-
piperonal	hexadecanoic acid, [2-phenyl-1,3-dioxolan-4-yl]methyl ester, cis-	cyclooctasiloxane, hexadecamethyl-	–	–	benzoic acid,4-hydroxy-2,6- dimethoxy-, methyl ester
2*H*-1-benzopyran-2-one,3,4-dihydro-	2-propanol, 1,1’-oxybis-	cyclodecasiloxane, eicosamethyl-	–	–	benzene, 4-(dimethoxymethyl)-1,2-dimethoxy-
vanillin	hexadecanoic acid, methyl ester	octasiloxane, 1,1,3,3,5,5,7,7,9,9,11,1,13,13,15,15 -hexadecamethyloctasiloxane	–	–	phenol, 2,4-bis(1,1-dimethylethyl)-
ethyl vanillin	octadecanoic acid, (2-phenyl-1,3-dioxolan,4-yl)methyl ester, cis-	heptasiloxane, 1,1,3,3,5,5,7,7,9,9,11,11,13,13-tetradecamethyl-	–	–	desulphosinigrin
2(3*H*)-furanone, 5-hexyldihydro-	diphenyl sulfone	–	–	–	dithiocarbamate,5- methyl-,*N*-(2-methyl-3-oxobutyl)-
benzaldehyde, 3,4-dimethoxy-	heptacosane	–	–	–	phenol, 3,5-bis(1,1-dimethylethyl)-
oxime-, methoxy-phenyl-	hexadecanoic acid, methyl ester	–	–	–	2-benzoyl -8-octanelactam
2(3*H*)furanone,dihydro-5-pentyl-	2-myristynoyl pantetheine	–	–	–	teredphthalic acid, 2-nitro-5-sulfanyl-
DL-xylitol, 1-benzoate	benzyl alcohol	–	–	–	–
sorbitol	1,2,3-propanetriol, diacetate	–	–	–	–
sulfide, sec-butyl isopropyl-	stearic acid, 3(octadecyloxy)propyl ester	–	–	–	–
	1,3-benzodioxole,5-(4-methyl-1,3-dioxolan-2-yl)-	–	–	–	–
	ethyl citrate	–	–	–	–
	benzoic acid, pentadecyl ester	–	–	–	–
	benzoic acid	–	–	–	–
	benzoic acid, hexadecyl ester	–	–	–	–

## Discussion

It is well understood that cigarette smoking can drive premature aging of the lungs, vasculature, and skin (Rashid et al., 2018; Morita, 2007). Chronic low-level inflammation and dysregulated ECM remodeling are common features in aged individuals and cigarette smokers are more likely to exhibit these features earlier compared to non-smokers (Csiszar et al., 2009; Sundar et al., 2016). Studies show that chronic cigarette smoke exposure impairs autophagy and proteostasis, leading to abnormal lung function (Tran et al., 2015; Bodas et al., 2016). Additionally, the combustion products of tobacco smoke generate oxidative stress and inflammation that inhibit collagen biosynthesis by skin fibroblasts leading to excessive wrinkling (Koh et al., 2002; Morita, 2007). While much is known about the effects of cigarette smoke and nicotine in aging, the effect of e-cigarettes on premature aging is poorly understood. In this study, we demonstrated that direct exposure to a mixed flavored e-liquid causes changes in cellular homeostasis consistent with accelerated aging observed in long time cigarette smokers. Exposure to the mixed flavored e-liquid induced inflammation, senescence and inhibited wound healing responses in pulmonary fibroblasts. This demonstrates that e-liquid exposure may have negative consequences on human health and may promote changes in cellular function associated with aging.

We assessed the cellular responses of pulmonary fibroblasts to direct e-liquid exposure. Cytotoxicity was observed at 0.5% concentration, but not with the concentration equivalent nicotine and PG/VG controls, suggesting that other constituents, such as flavoring chemicals, are responsible for cell death. This is consistent with other studies that show cytotoxicity with flavoring compounds, independent of other e-cigarette components such as PG/VG and nicotine (Bitzer et al., 2018; Muthumalage et al., 2019).

To determine how e-liquid exposure may exacerbate aging, we looked at the release of inflammatory mediators and the development of senescence. The number of senescent fibroblasts increase in older individuals and patients with COPD. They also show increased levels of inflammatory mediators such as interleukin-8 (IL-8), prostaglandin E2 (PGE_2_), and interleukin-6 (IL-6) (Larsson, 2008; Zhang et al., 2012). Acute e-liquid exposure resulted in increased IL-8 release after 24 h. Higher doses failed to induce IL-8 secretion, which may be a consequence of increased cytotoxicity. IL-8 is a potent chemokine for neutrophils and plays an important role in sustaining chronic inflammation (Reynolds et al., 2018). In our previous work, tobacco-flavored e-cigarettes failed to elicit an inflammatory response in monocytes and fibroblasts, in contrast to what we have observed with this tobacco flavored e-liquid (Lerner et al., 2015b; Muthumalage et al., 2017). However, e-liquids represent a mixture of chemicals and interactions between tobacco flavors with other flavors may alter cellular responses. Moreover, e-liquid and PG/VG exposure increased cellular senescence, which may perpetuate inflammatory responses through SASP.

Prolonged inflammation and oxidative stress initiate the reorganization of the extracellular matrix. The ECM plays a vital role in injury responses (Crotty Alexander et al., 2018). Unresolved damage to the lung can perturb the normal wound healing process, which is observed in ILD and increases with age (Gould et al., 2015). In this study, the treatment of pulmonary fibroblasts with e-liquid did not significantly alter the production of fibronectin or collagen. However, we observed inhibition of myofibroblast differentiation, suggesting that this e-liquid may potentially inhibit wound healing responses in the lung. TGF-β1-induced fibronectin was also significantly inhibited with e-liquid exposure. Previously, we have reported that nicotine reduces the wound healing capacity by inhibiting contraction and myofibroblast differentiation. Nicotine inhibits myofibroblast differentiation by interfering with mitochondrial dynamics and these effects can be recapitulated with mitochondrial complex II inhibitor antimycin A (Lei et al., 2017). However, further studies would need to assess if other constituents besides nicotine are playing a role. In contrast to patients with ILD, we observed a decrease in ECM production. Fibroblasts are a heterogeneous population and the effects of premature senescence on different populations may differentially affect deposition and resolution phases in wound healing (Waters et al., 2018). Stress-induced senescence in progenitor populations may also negatively affect the ability of these cells to respond to injury. In addition, senesced fibroblasts secrete more matrix metalloproteases and limit fibrosis under certain conditions (Krizhanovsky et al., 2008; Li et al., 2016), which could prevent proper remodeling of the ECM. Disruption of collagen biosynthesis may also cause advanced aging of the skin. However, further research in skin fibroblasts is needed to make any conclusions.

We have previously demonstrated that mixed e-liquid flavors induce more severe cytotoxicity, generation of ROS, and inflammatory responses in comparison to a single flavor suggesting that mixing of flavors form secondary products eliciting an exacerbated cellular response (Muthumalage et al., 2017). The key constituents were identified through GC-MS analysis, which revealed that pyrazines, vanillin, and furonones, all known pulmonary irritants, were some of the main flavoring constituents present in this e-liquid. Pyrazines are associated with chocolate or roasted nut flavors. Pyrazines contain a heterocyclic motif that interacts with a diverse set of targets such as p53, the estrogen receptor, and the vascular endothelial growth factor (VEGF) making them an attractive target in the treatment of multiple diseases such as various cancers (Browne et al., 1991; Kamal et al., 2011; Lalitha et al., 2016). In hepatic stellate cells, tetramethylpyrazine induced senescence through a p53 dependent mechanism (Jin et al., 2017). Vanillin was associated with higher cytotoxicity in high throughput screening assays (Sassano et al., 2018). In addition, aldehydes like vanillin generate oxidative stress and are known to activate DNA damage responses (Sundar et al., 2016). Furan and its derivatives, which are often found in fruity and sweet flavors, are associated with damage to nasal mucosa and the lamia propia in rats (Arts et al., 2004). These compounds also exhibited anti-cancer properties in a lung adenocarcinoma cancer line (Yuan et al., 2006; Byczek-Wyrostek et al., 2018). The presence of silicon oils (siloxanes) is also a cause for concern. Inhalation of high concentrations of silicon compounds can lead to respiratory irritation, leukocytosis, and may contribute to the development of pulmonary edema and lesions (Jean and Plotzke, 2017; Muthumalage et al., 2020). While the qualitative nature of this data precludes us from making stronger associations, this data shows some evidence of exposure to chemicals capable of inducing cell growth arrest and senescence.

Our study has some limitations that need to be considered. We exposed fibroblasts to the e-liquid directly rather than exposure to aerosolized e-liquid. This does not consider the possibility that combustion products may form during heating and aerosolization of the e-liquid that may affect the toxicity outcomes we observed. Secondly, we conducted acute exposures, when the contributions of e-cigs to the pathogenesis of ILDs and aging would likely occur over extended periods of time. Furthermore, we tested only one mixed flavored e-liquid as shown above, whereas other flavor combinations are available commercially which need to be tested in order to evaluate the effects of various mixed flavors on biological systems.

In conclusion, e-liquids containing multiple flavors are more toxic and induces an exacerbated cellular response in comparison to single flavors. Thus, identifying the responsible flavoring chemicals that play a role in lung disease is vital for the regulation of flavors and their constituents. Considering the recent federal ban on flavored e-cigarettes, it is important to consider which ingredients represent the greatest health hazard as consumers look to other sources for flavored nicotine products. GC-MS analysis of the flavored e-liquid revealed the presence of known cytotoxic constituents that implicate it in age associated chronic lung injury. E-liquid exposure also caused inflammation and cellular senescence in pulmonary fibroblasts along with inhibition of myofibroblast differentiation and ECM production. Premature aging of the lung and skin may be a consequence of dysregulated ECM remodeling in senescent fibroblasts. However, further work must be conducted *in vivo* and with skin fibroblast to assess the crosstalk between these two processes. That aside, these data indicate that inhalation of e-liquids poses a health concern and that further regulation is required for the main chemicals identified in e-liquid flavors.

## Data Availability Statement

The original contributions presented in the study are included in the supplementary material. Further inquiries can be directed to the corresponding author(s).

## Author Contributions

JL, TM, and IR conceived and designed the experiments. JL and TM conducted the experiments. JL wrote the manuscript. JL, TM, QW, and IR edited the manuscript. MF and AF analyzed the chemistry data. All authors contributed to the article and approved the submitted version.

## Conflict of Interest

The authors declare that the research was conducted in the absence of any commercial or financial relationships that could be construed as a potential conflict of interest.

## Footnotes

1 xtremevaping.com
